# First description of ultramutated endometrial cancer caused by germline loss-of-function and somatic exonuclease domain mutations in *POLE* gene

**DOI:** 10.1590/1678-4685-GMB-2020-0100

**Published:** 2020-09-25

**Authors:** Reginaldo Cruz Alves Rosa, Andrey A. Yurchenko, Fernando Chahud, Alfredo Ribeiro-Silva, Mariângela Ottoboni Brunaldi, Wilson Araújo Silva, Patricia L. Kannouche, Sergey Nikolaev, Victor Evangelista de Faria Ferraz

**Affiliations:** 1 Universidade de São Paulo, Faculdade de Medicina de Ribeirão Preto, Departamento de Genética, Ribeirão Preto, SP, Brazil.; 2 Université Paris Saclay, Inserm U981, Gustave Roussy Cancer Campus, Villejuif, France.; 3 Universidade de São Paulo, Faculdade de Medicina de Ribeirão Preto, Departamento de Patologia e Medicina Legal, Ribeirão Preto, SP, Brazil.; 4 Paris-Sud University, CNRS-UMR 8200, Equipe labellisée Ligue Contre le Cancer, Gustave Roussy Cancer Campus, Villejuif, France.; 5 Universidade de São Paulo, Centro de Genômica Médica, Hospital das Clínicas, Faculdade de Medicina de Ribeirão Preto, Ribeirão Preto, SP, Brazil.

**Keywords:** Endometrial cancer, *POLE* exonuclease mutation, Targeted sequencing, TMB, Ultramutated phenotype

## Abstract

Endometrial cancer (EC) harboring heterozygous *POLE* proofreading inactivating mutations (*POLE*-exo*) is associated with an increased number of somatic mutations that result in a distinctive anti-tumor immune response. However, the consequences of such *POLE* mutations in the context of the missing wild-type allele have not yet been described in endometrial tumors. A 72-year-old woman harboring a germline monoallelic frameshift mutation (p.Pro269fsTer26) in *POLE* was diagnosed with an EC having a somatic heterozygous mutation in the exonuclease domain of *POLE* (S459F). Targeted gene sequencing revealed an ultramutated phenotype (381 mutations/Mb) in the tumor and a 2-fold excess of mutations on the DNA leading strand. Additionally, we observed a mutational signature similar to the COSMIC signature 10, a higher mutation rate in this tumor than in endometrial tumors with heterozygous *POLE*-exo*, and an increased number of T lymphocytes. This is the first report of an ultramutated EC harboring a somatic *POLE*-exo* mutation in association with a germline loss-of-function mutation in this gene. The absence of a wild type *POLE* allele led to a particularly high mutational burden.

Endometrial cancer (EC) is a heterogeneous malignancy characterized by several different histologic subtypes with endometrioid carcinoma being the most common (McConechy *et al.*, 2016). Recently, there have been significant advances in defining the molecular alterations that contribute to tumorigenesis in EC. The Cancer Genome Atlas Research Network (TCGA) divides EC into four categories based on recurrent molecular features: an ultramutated phenotype caused by *POLE* mutations, a hypermutator phenotype caused by the DNA mismatch repair deficiency (MMRD) leading to microsatellite instability (MSI), a copy number low phenotype, and a copy number high phenotype (Levine *et al.*, 2013).

The *POLE* gene encodes the catalytic subunit of DNA polymerase ε (Pol ε), which replicates the leading strand during DNA replication (Burgers *et al.*, 2017). In addition to DNA-binding and polymerase domains, Pol ε has proofreading activity through its exonuclease domain. This capacity is essential for the maintenance of replication fidelity, and this proofreading function may act, not only on newly misincorporated nucleotides, but also on mismatches produced by non-proofreading polymerases (Palles *et al.*, 2013). Up to 12% of all endometrial carcinomas harbor *POLE* mutations that tend to cluster in the exonuclease domain (*POLE*-exo*), especially in the conserved residues 268 to 471 (Billingsley *et al.*, 2016; Bellone *et al.*, 2017; Barbari *et al.*, 2018). Tumors harboring such mutations are associated with an ultramutated phenotype, increased neoantigen load, increased tumor infiltrating lymphocytes, and increased potential for responding to immunotherapy (Imboden *et al.*, 2019).

Germline mutations in the exonuclease domain of *POLE* are infrequent; most *POLE*-exo* mutations are somatic and occur in sporadic tumors almost exclusively in a heterozygous state because their dominant nature (Wong *et al.*, 2016; Barbari *et al.*, 2017). Additionally, there is no associated *POLE* inactivation by somatic loss of heterozygosity (LOH) when tumors occur in carriers of germline *POLE* mutations (Palles *et al.*, 2013). However, a minority of tumors with *POLE*-exo* show LOH or other inactivating mutations that could act as ‘second hits’ (Heitzer *et al.*, 2014). Curiously, loss or inactivation of the second allele has been reported in a few colorectal tumors with mutations disturbing Pol ε proofreading activity and at least one example illustrates that this mutation may have phenotypic consequences for disease presentation (Muzny *et al.*, 2012). However, similar findings have not been reported for endometrial tumors (Shinbrot *et al.*, 2014).

Here, we report a 72-year-old woman diagnosed with a FIGO Grade 1 and FIGO Stage 1B endometrial endometrioid adenocarcinoma at 63 years old. A total hysterectomy and salpingo-oophorectomy were performed. The patient reported no familial history of cancer. Immunohistochemistry (IHC) of the MMR proteins and MSI analysis were performed. The tumor had an intact expression of MLH1, MSH2, MSH6 and PMS2 proteins, based on immunohistochemical analysis, and was classified as MSI-low based on the MSI assay. A germline and somatic mutation screening were performed, and the mutational profile and its immunologic characterization of the endometrial tumor were accessed (for details of material and methods, see Mat-Met S1 in Supplementary Material). The study was approved by the Scientific and Research Committee of the Clinics Hospital of the Ribeirão Preto Medical School (protocol number: 1.578.206). Informed written consent was obtained from the patient.

For germline mutation screening, a targeted sequencing assay of the coding, canonical splice sites, and both 5’ and 3’ untranslated regions of 63 genes (Table S1), including Lynch syndrome-associated genes and *POLE,* was performed in DNA extracted from peripheral blood. Single nucleotide variants (SNVs) and Copy number variation (CNV) were evaluated. Only the germline frameshift mutation NM_006231:c.806delC (p.Pro269fsTer26) in *POLE* was identified (Figure 1A), with a variant allele frequency (VAF) of 0.50 (total coverage = 729 reads), as expected for a heterozygous germline variant.

**Figure 1:**
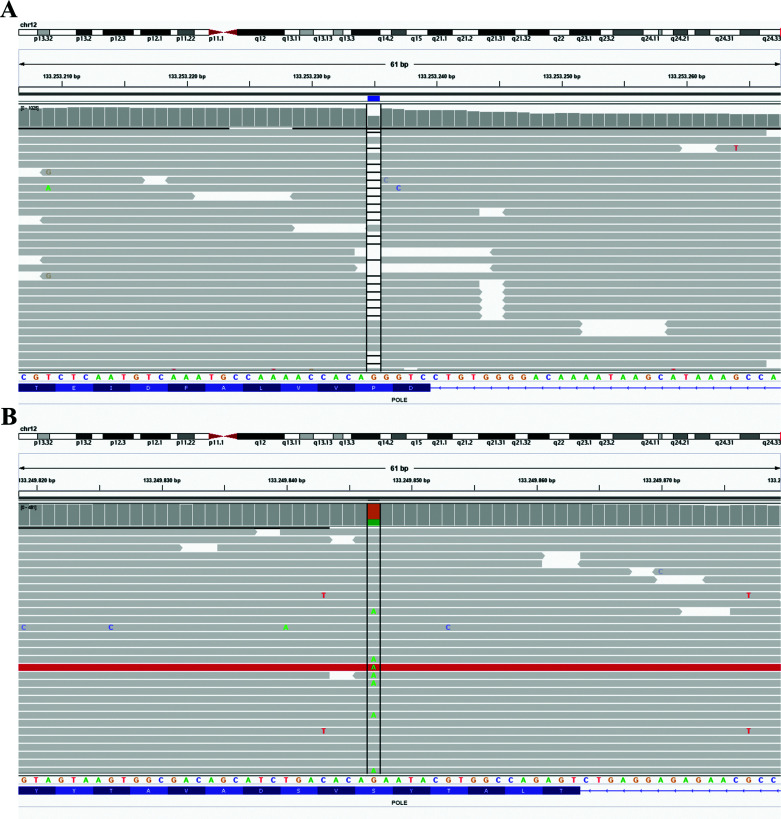
Integrative Genomics Viewer snapshot of *POLE* mutations with reference *POLE* nucleotide and amino acid sequences. **(A)** germline frameshift c.806delC and **(B)** somatic c.1376C>T (S459F) exonuclease mutation.

Since this germline mutation could not explain the tumor MSI-low phenotype present in the EC, a further mutational search was performed using the tumor DNA. For somatic analysis, targeted sequencing using the same gene panel described for germline analysis was performed on genomic DNA extracted from a representative tumor area (at least 70% of tumor cells) from Formalin-Fixed Paraffin-Embedded (FFPE) blocks. Both somatic SNVs and CNVs were called on the matching tumor-blood DNA samples. As expected, the germline frameshift mutation in *POLE* was also detected in tumor sequencing, with a VAF = 0.51 (total coverage = 242 reads), supporting its germline origin. Additionally, a somatic mutation in the exonuclease domain of *POLE*, S459F (NM_006231:c.1376C>T, p.Ser459Phe), was observed with VAF = 0.298 (Figure 1B). As long as this mutation is heterozygous, it is expected to be present in ~60% of cells in the tumor sample, these estimates are based on a tumor purity of 80% from the histological examination. We did not find any pathogenic mutation neither in the MMR genes (*MLH1*, *MSH2*, *MSH6*, and *PMS2*) not in the exonuclease domain of *POLD1*.

The tumor mutational profile was investigated to determine whether the genomic alterations were consistent with a *POLE* ultramutator phenotype. A total of 190 mutations were identified in the sequenced region of the 63 gene panel (0.49 Mb). Considering only the coding region, 0.257 Mb distributed along 937 exons of 63 cancer-related genes, a total of 95 mutations were identified, resulting in a mutation rate of 381 mutations/Mb. A total of 65 nonsynonymous mutations were identified in the targeted exons, resulting in an estimated tumor mutation burden (TMB) of 253 nonsynonymous mutations/Mb.

The trinucleotide context of mutations was investigated, and a mutational signature analysis was performed using the database of the known mutational signatures in human cancers from Alexandrov *et al.* (2013). Given the high number of somatic mutations identified, we had sufficient data to derive a mutational signature that was closely related to COSMIC signature 10 (Cosine similarity = 0.97, Figure 2A). These findings are indicative of mutations in DNA replication associated with errors in proofreading activity of Pol ε. Most nucleotide substitutions detected in the tumor sample were represented by C>A, C>T, and T>G, with a relative contribution to the total amount of substitution mutations of 0.43, 0.33 and 0.18, respectively (Figure 2B).

**Figure 2:**
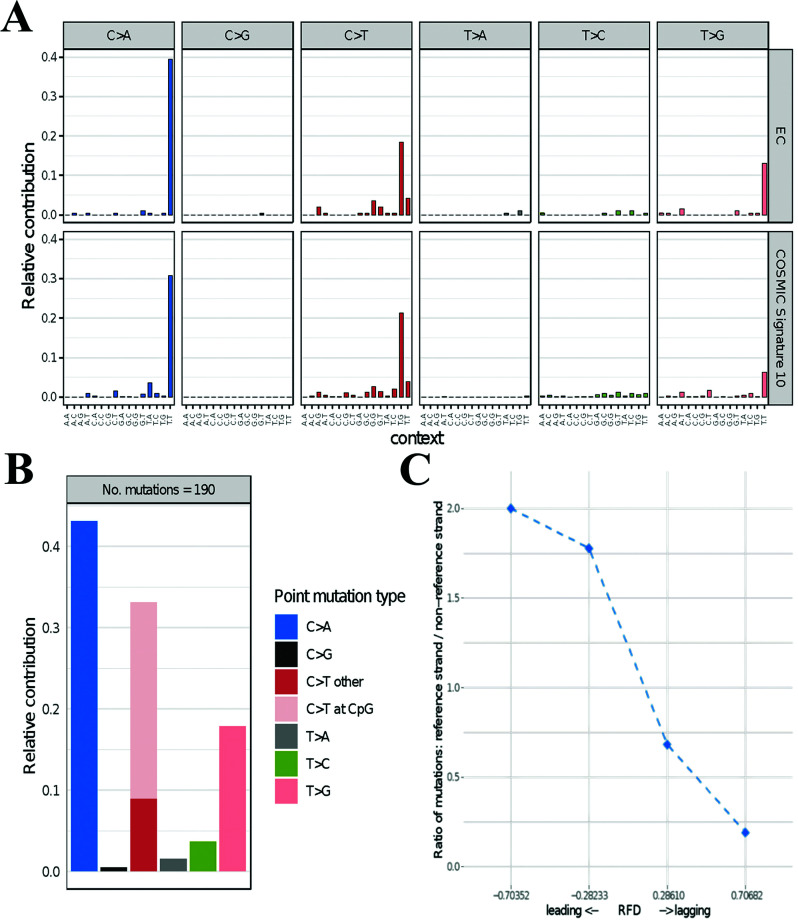
Mutational profile based on targeted sequencing data of a 63 cancer-related gene panel. **(A)** mutational signature extracted from the endometrial carcinoma in comparison to the COSMIC signature 10. **(B)** relative contribution of each point mutation type to the total amount of somatic mutations. **(C)** Strand bias analysis showing a predominance of mutations on the leading strand. EC, endometrial cancer.

In addition to mutational signature analysis, we calculated the mutation strand bias asymmetry between the leading and lagging DNA strands. There was a 2-fold excess of mutations on the leading strand of DNA in comparison with the lagging strand (Figure 2C). These molecular findings highlight the strong effect of defects in Pol ε proofreading activity in this reported EC.

In order to compare the mutation rate and total TMB between the studied tumor and endometrial cancers with heterozygous *POLE*-exo* mutations, we downloaded 25 exomes of endometrial carcinoma from ICGC portal with *POLE*-exo* somatic mutations and absence of MSI (Zhang *et al.*, 2011). To minimize the influence of different capture kits we estimated TMB only in the coding regions of our gene panel. The mutation rate observed in the endometrial carcinoma reported here (381 mutations/Mb) is more than 2-fold higher than the average rate observed in 25 endometrial carcinomas harboring heterozygous *POLE*-exo* mutations (153 mutations/Mb, ranging from 47 to 276). One out of 25 endometrial carcinomas harbored the heterozygous *POLE*-exo* S459F and presented a rate of 167 mutations/Mb (Table 1). These data confirm that EC harboring a LoF genetic event in *POLE* in association with a heterozygous *POLE*-exo* mutation confers an augmented mutator phenotype in comparison with EC with single *POLE*-exo* alterations.

**Table 1:** Mutational profile of endometrial carcinomas harboring heterozygous *POLE*-exo* mutations in comparison with the endometrial cancer reported.

Sample	*POLE*-exo* mutation	Total amount of mutations^a^	Mutations/Mb	Nonsynonymous mutations	TMB^b^
Report	S459F	95	381	65	253
SA485042	P286R	71	276	53	206
SA475378	P286R	70	272	47	183
SA552345	P286R	69	268	44	171
SA472709	P286R	59	230	42	163
SA466958	P286R	61	237	41	160
SA470974	P286R	64	249	41	160
SA462048	P286R	63	245	39	152
SA467568	P286R	47	183	35	136
SA483959	P286R	48	187	32	125
SA541518	P286R	39	152	29	113
SA472897	S459F	43	167	29	113
SA20267	P286R	38	148	27	105
SA561528	P286R	49	191	24	93
SA469202	P286R	31	121	20	78
SA473549	P286R	26	101	20	78
SA526095	P286R	28	109	18	70
SA541610	P286R	24	93	18	70
SA526120	P286R	26	101	15	58
SA92158	P286R	29	113	15	58
SA482148	P286R	24	93	13	51
SA479614	P286R	19	74	12	47
SA467039	V411L	16	62	10	39
SA476079	V411L	15	58	9	35
SA474561	P286R	12	47	7	27
SA519177	P286R	12	47	6	23

a all mutations were identified along the 0.257 Mb of the 63 gene panel, including synonymous and nonsynonymous mutations.

b expressed as number of nonsynonymous mutations/Mb. TMB, tumor mutational burden. Report: endometrial cancer case characterized in the present study.

For evaluation of tumor-associated lymphocytes, the mean number of CD3+, CD4+, and CD8+ of intraepithelial T lymphocytes, i.e., T lymphocytes located within the tumor epithelium was calculated. IHC staining for T lymphocyte markers revealed a predominance of CD8+ lymphocytes in the intra-tumoral area in comparison with CD4+ T cells, with mean numbers of 29.9 CD8+, and 10.9 CD4+ T-cells. A mild (1+) presence of CD3+, CD4+, and CD8+ lymphocytes was observed in the peri-tumoral region (Figure 3).

**Figure 3:**
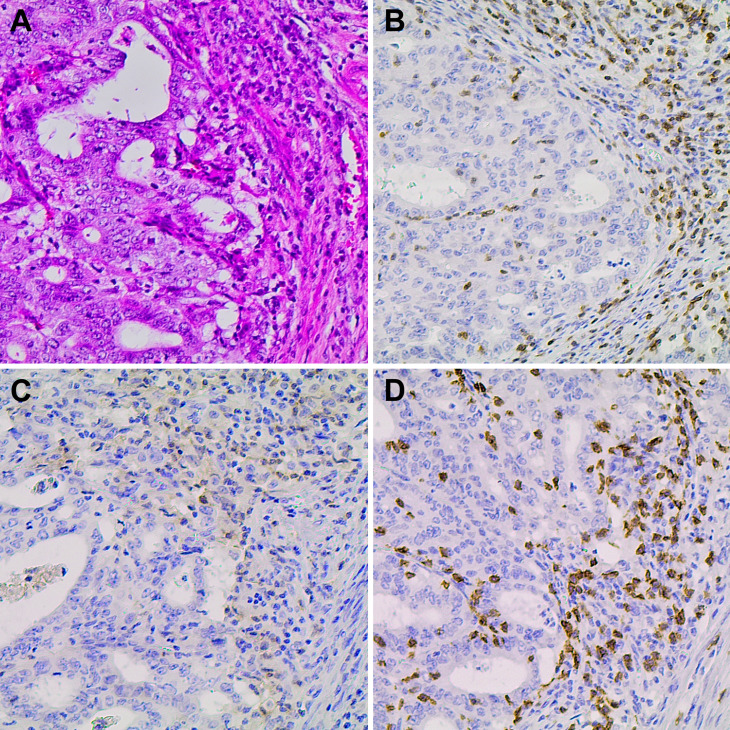
Immunohistochemical staining for T lymphocyte markers on the peri and intra-tumoral areas of the EC (original magnification x200) . **(A)** H&E (hematoxilin-eosin). Brown nuclear staining is indicative of positive expression **(B)** CD3, **(C)** CD4 and **(D)** CD8 markers.

This is the first case of an endometrial carcinoma harboring a somatic *POLE* exonuclease mutation related to an ultra-mutator phenotype acting as a ‘second hit’ in association with a germline truncating mutation of the gene. Germline heterozygous missense mutations affecting the *POLE* exonuclease domain are associated with a syndrome called Polymerase Proofreading-Associated Polyposis (PPAP) that increases the risk for the development of multiple colorectal adenomas and colorectal cancer (Briggs *et al.*, 2013). A diagnosis of PPAP is not consistent with findings in the patient presented in this case report since she carries a germline frameshift mutation at the beginning of the *POLE* exonuclease domain that creates a premature termination codon. Truncating mutations in *POLE* gene are unlikely to lead to PPAP phenotype, since a successful DNA synthesis must occur before the proofreading activity of Pol ε (Heitzer *et al.*, 2014). However, the co-occurrence of a germline truncating mutation with a somatic ultra-mutator phenotype-associated variant in *POLE* suggests a complete loss of Pol ε proofreading activity in the endometrial tumor. Thus, by itself the germline frameshift mutation does not confer a genetic predisposition to EC and cannot lead to a mutator phenotype in the tumor, but might contribute to increase the mutational load because only proofreading-deficient Pol-ε will replicate DNA in these tumor cells.

Some *POLE*-exo* mutations have been described to be associated with an ultra-mutator phenotype, with varying levels of mutation. Previous functional studies have demonstrated the exonuclease deficiency effect of the *POLE* S459F mutation *in vitro* (Shinbrot *et al.*, 2014) as well as the moderate mutator effect in yeast (Barbari *et al.*, 2018). The EC reported here was MSI-low. Co-occurrence of MSI and *POLE*-exo* mutations, usually with the P286R mutation, in endometrial tumors has already been reported (Haradhvala *et al.*, 2018). However, all tumors described to date that harbor the S459F mutation in *POLE* were found to be microsatellite stable (Shinbrot *et al.*, 2014; Andrianova *et al.*, 2017; Barbari *et al.*, 2018). Our study is the first report of a tumor harboring the S459F mutation this is associated with an MSI-low phenotype.

Somatic mutations found in cancer genomes are the consequence of the intrinsic infidelity of the DNA replication machinery, exogenous or endogenous mutagen exposures, enzymatic modification of DNA, or defective DNA repair and other processes. Different mutational processes often generate variation in the combinations of mutation types, termed mutational signatures (Alexandrov *et al.*, 2013). More than 30 mutational signatures have already been identified across 40 different types of human cancer (Forbes *et al.*, 2017). We identified a mutational signature that closely resembles the COSMIC signature 10, which is known to be associated with *POLE*-exo* mutations (Alexandrov *et al.*, 2013). The *POLE* mutational signature is characterized by a 100-fold increase in C>A transversions in the context TCT and a 30-fold increase in C>T transitions in the context TCG (Rayner *et al.*, 2016). This mutational pattern results in a strong bias for particular amino acid changes, with an overrepresentation of serine to tyrosine or leucine, and arginine to isoleucine or glutamine substitutions, and a substantial increase in glutamic acid to stop codon mutations (Rayner *et al.*, 2016). Although mutational signatures are preferably determined by genomic analysis, such as whole genome sequencing (WGS) and whole exome sequencing (WES), we were able to identify a mutational signature related to *POLE*-exo* mutations through targeted sequencing of the coding and regulatory regions of only 63 genes. These findings support that mutational signatures can be extracted from sequencing data derived from a small gene panel in tumors that are highly mutated (Hoeck *et al.*, 2019). In addition, we observed a strong strand bias effect with mutations occurring predominantly in the leading strand in comparison with the lagging strand. This phenomenon, in addition to the mutational signature close to COSMIC signature 10, highlights the major effect of *POLE* proofreading inactivation in the EC reported here.

TMB is a quantitative measure of the total number of somatic nonsynonymous mutations per coding area of a tumor genome and is associated with the emergence of neoantigens that trigger anti-tumor immunity (Allgäuer *et al.*, 2018; Meléndez *et al.*, 2018). We identified a total of 65 nonsynonymous mutations along 0.257 Mb coding regions of the sequenced gene panel, resulting in an estimated TMB of 253 nonsynonymous mutations/Mb. Although a wider genomic analysis is required to achieve the precise TMB (Büttner *et al.*, 2019), the absolute amount of somatic nonsynonymous mutations (65 mutations/0.257 Mbp) observed in the EC reported here is superior to the threshold of 20 mutations/Mb commonly used to classify a tumor with high TMB and as an immunotherapy responder (Allgäuer *et al.*, 2018; Endris *et al.*, 2019). The absolute amount of nonsynonymous mutations, as well as the total number of mutations (95 mutations/0.257) identified in our report is higher than those identified in EC harboring heterozygous *POLE*-exo* mutations. We used the GATK pipeline, which has a high sensitivity and specificity for somatic mutations calling and checked the occurrence of FFPE-derived artefacts in the sequencing data. Thus, the higher mutational load identified in the endometrial tumor in comparison with the ICGC/TCGA Pole-exo* tumors is not supposed to be led by interstudy differences.

The occurrence of two proofreading-inactivating events in *POLE* is extremely rare, suggesting that *POLE* may not act as a classical tumor suppressor gene (Heitzer *et al.*, 2014). There is a single case of colorectal cancer (CRC) in The Cancer Genome Atlas (TCGA) project carrying the *POLE* S459F mutation and a nonsense mutation at codon 150 of the *POLE* gene, which was thought to inactivate the second allele (Muzny *et al.*, 2012). This CRC presented a higher number of somatic mutations (376 mutations/Mb) in comparison with another TCGA-CRC harboring only the S459F mutation in heterozygosity (81 mutations/Mb) (Shinbrot *et al.*, 2014). Both mutations present in the TCGA-CRC with two hits in *POLE* are somatic. Our findings are novel since we report an endometrial carcinoma harboring one germline *POLE* LoF mutation and one somatic *POLE*-exo* mutation.

Molecular classification of human cancer represents an important step toward the goal of precision medicine and helps to identify patients who would benefit from targeted immunotherapy (Liu *et al.*, 2019). We observed the occurrence of a greater number of CD8+ T lymphocytes in comparison with CD4+ T-cells in the peri and intra-tumoral area in our EC case. *POLE*-exo* mutations have been associated with increased tumor infiltrating lymphocytes, especially CD8+ (Howitt *et al.*, 2015; Bourdais *et al.*, 2017).

The characterization of the mutational pattern, as well as the lymphocyte profile revealed an accentuated Pol ε proof-reading failure in an EC harboring a germline and a somatic mutation at the *POLE* exonuclease domain. These findings suggest that the mutations are in trans, *i.e*. located in different DNA strands. The frameshift mutation affects the beginning of the exonuclease domain of *POLE* and is expected to result in a truncated, immature, or non-functional protein. If the *POLE* S459F mutation were located at the same strand as the germline frameshift, the ultramutator effect would likely be silenced by the frameshift. However, as a limitation of our study, we could not experimentally prove that the frameshift and missense *POLE*-exo* mutations are in trans and neither that the frameshift indeed led to the silencing of one *POLE* allele, due to the high fragmentation of DNA and RNA derived from FFPE slides. Additionally, although we have strong evidence supporting that our EC case has a higher mutational load identified in comparison with tumors harboring heterozygous *POLE*-exo* mutations, we are aware that the number of mutations identified might have been affected by interstudy differences in sample preservation methods, library protocols, and bioinformatic pipelines. Also, although we observed a higher mutational load in the EC with two genetic events at the proof-reading domains of *POLE* in comparison with EC harboring only a heterozygous *POLE*-exo* mutation, we would need to have more tumors with similar findings in order to make statistically significant conclusions about the mutational burden of these tumors relative to the cancers with heterozygous *POLE*-exo* mutations.

In conclusion, our EC case exhibits molecular and histopathological features typically linked to *POLE* exonuclease mutated tumors. The comparison with other tumors with *POLE*-exo* mutations suggests that the absence of the wild type *POLE* allele renders particularly higher TMB in such tumors. Consequently, detection of a combination of *POLE*-exo* and LoF *POLE* mutations could be considered as prognostic or therapeutic marker.

## Supplementary material

The following online material is available for this article:

Mat-Met S1 Detailed description of all material and methods used in this study.

Table S1 Detailed list of the 63 genes used for targeted sequencing.
