# Supplementary material for: First description of ultramutated endometrial cancer caused by germline loss-of-function and somatic exonuclease domain mutations in POLE gene
Source: Genet Mol Biol. 2020 Sep 25;43(4):e20200100. doi: 10.1590/1678-4685-GMB-2020-0100 (PMC7521106; doi:10.1590/1678-4685-GMB-2020-0100)
Supplement: Supplementary file 1 [file 1415-4757-GMB-43-4-e20200100-suppl1.pdf]

Genetics and Molecular Biology
https://doi.org/10.1590/1678-4685-GMB-2020-0100

Supplementary Material to "First description of ultramutated endometrial cancer caused by germline loss-of-function and somatic exonuclease domain mutations in *POLE* gene"

Mat Met S1 - Detailed Material and Methods

Immunohistochemistry (IHC)

IHC staining of MLH1, MSH2, MSH6, PMS2, CD3, CD4, and CD8 was performed on 4 µm sections of formalin-fixed and paraffin-embedded (FFPE) tumoral tissue according to standard procedure. The primary antibodies were anti-MLH1 (dilution 1:100; clone 6168-728; BioSB, Santa Barbara, CA), anti-MSH2 (dilution 1:250; clone 25D12; Leica Biosystems, Buffalo Grove, IL), anti-MSH6 (dilution 1:75; clone 6TBP H-141; Santa Cruz Biotechnology, Santa Cruz, CA), anti-PMS2 (dilution 1: 25; clone MOR46; Leica Biosystems, Buffalo Grove, IL), anti-CD3(dilution 1:200; clone PS1; Neomarkers, Fremont, CA), anti-CD4 (dilution 1:50; clone 4F12; Dako, Santa Clara, CA), and anti-CD8 (dilution 1:200; Novocastra, Buffalo Grove, IL). The analysis was performed by two independent pathologists (FC and ARS). For MMR protein evaluation, adjacent normal endometrium or lymphocytes in the slides were used as an internal positive control and loss of MMR protein expression was defined as the complete absence of nuclear staining in all tumor cells. For evaluation of tumor-associated lymphocytes, the mean number of CD3+, CD4+, and CD8+ of intraepithelial T lymphocytes, i.e., T lymphocytes located within the tumor epithelium, rather than in the peritumoral stroma, was calculated from photomicrographs (40X objective) of 10 high-power fields (HPFs). Peritumoral T lymphocytes (T lymphocytes in the stroma immediately adjacent to the tumor epithelium) were scored using a semiquantitative method (none (0), mild (1+), moderate (2+), marked (3+)) as described by Howitt *et al.* (2015).

Microsatellite instability (MSI)

The pathologists (A.R.S., F.C., and M.O.B.) manually inspected the H&E slides in order to delimitate tumor and non-tumor areas from each case. Non-neoplastic adjacent uterine areas were used as normal tissue source. Genomic DNA from tumor and normal tissues were extracted from FFPE sections using the Maxwell Rapid Sample Concentrator System (Promega, Madison – WI). MSI status was performed using multiplexed polymerase chain reaction (PCR) for genotyping the monomorphic repetitive markers: BAT26, BAT25, NR21, NR24, and NR27. Primer sequences and PCR conditions were described elsewhere (Buhard *et al*., 2004). Amplicon detection and analysis were performed using an ABI Prism 3130 Genetic Analyzer (Applied Biosystems, Foster City, CA) and GeneMarker v.1.85 (SoftGenetics, State College, PA), respectively. A diagnosis of MSI-high (MSI-H) was considered positive when two or more markers showed an altered pattern; MSI-low (MSI-L) when one marker was altered and microsatellite stable (MSS) when none of the markers showed alteration.

Targeted sequencing

For germline analysis, peripheral blood was extracted using QIAamp DNA Mini Kit (Qiagen, Germantown, MD) following manufacturer's instructions and library preparation was performed using the SureSelectQXT Kit (Agilent Technologies, Santa Clara, CA). For somatic analysis, genomic DNA was extracted from a representative tumor area (at least 70% of tumor cells) from FFPE slides, and the library was prepared using the SureSelectXT Kit (Agilent Technologies). For both germline and somatic mutation analysis, the coding, canonical splice sites, and both 5' and 3' untranslated regions (UTRs) of 63 genes (Supplementary Table S1), including Lynch syndrome-associated genes and *POLE*, were sequenced on an Illumina NextSeq 500/550 system (Illumina, San Diego, CA) on a 2 x 150 bp paired-end mode.

Bioinformatics Analysis

The raw FASTQ files were aligned to the human reference genome GRCh37 with BWA (Li and Durbin, 2010), sorted with Samtools (Li *et al*., 2009) and the resulted BAM files were further processed with GATK v. 4.0.10.1 (McKenna *et al*., 2010) to remove duplicated read pairs and recalibrate read

quality scores according to the GATK Best Practises protocol (Van der Auwera *et al*., 2013). The germline variants were identified using HaplotypeCaller in the BAM file generated from the blood DNA sample reads and then annotated using ANNOVAR software (Wang *et al*., 2010). Somatic variants were called on the matching tumour – blood DNA samples with Mutect2 algorithm (Cibulskis *et al*., 2013). After the annotation of somatic variants with Oncotator (Ramos *et al*., 2015) we left only variants with "PASS" flag and Variant Allele Frequency (VAF) higher than 5% to reduce the number of false-positive variants abundant in FFPE samples (Prentice et al., 2018). The final set of somatic variants was additionally annotated using OncoKB (Chakravarty *et al*., 2017) database to identify known cancer-driving events in the studied tumor. Copy-number alterations (CNV) in the tumor were assessed using FACETS software (Shen *et al*., 2017). VisCap was use for germline CNV investigation (Pugh *et al*., 2016).

Mutational signature analysis and Cosine similarity calculation were performed in R package Mutational Patterns (Blokzijl *et al*., 2018) using database of the known mutational signatures in human cancers from Alexandrov *et al*. (2013).

To calculate the strand bias asymmetry between the leading and lagging strands, we used a map of replication fork direction generated based on the Okazaki fragments sequencing and subsequent model segmentation from Petryk *et al*. (2016). The probability of replication fork direction in each 1 kb window was averaged between the HeLa and GM06990 cell lines, and only regions consistent between them were left for the analysis.

To compare mutation rate and tumor mutational burden (TMB) between the studied tumor and endometrial cancers with heterozygous *POLE*-exo* mutations we downloaded VCF files containing preexisting somatic mutation calls from 25 exomes of endometrial carcinomas from ICGC portal (https://dcc.icgc.org) with *POLE*-exo* heterozygous somatic mutations, *POLD1*-exo wild type, and absence of MSI (Zhang *et al*. 2011). We analyzed the same genomic regions in both the targeted sequencing and the WES data to compare mutational load between our EC case and ICGC data.

Sanger sequencing

https://doi.org/10.1590/1678-4685-GMB-2020-0100

For orthogonal validation, *POLE* variants were confirmed by Sanger sequencing performed on a 3500xl sequencer using the BigDye 3.1 sequencing kit (Applied Biosystems, Carlsbad, CA, USA). Primer sequences used were reported previously (Yoshida *et al*., 2011).

References

Alexandrov LB, Nik-Zainal S, Wedge DC, Aparicio SA, Behjati S, Biankin AV, Bignell GR, Bolli N, Borg A, Børresen-Dale AL *et al*. (2013) Signatures of mutational processes in human cancer. Nature 500:415-421.

Blokzijl F, Janssen R, van Boxtel R and Cuppen E (2018) MutationalPatterns: comprehensive genome-wide analysis of mutational processes. Genome Med. 10:33.

Buhard O, Suraweera N, Lectard A, Duval A and Hamelin R (2004) Quasimonomorphic mononucleotide repeats for high-level microsatellite instability analysis. Dis Markers. 20:251-257.

Chakravarty D, Gao J, Phillips SM, Kundra R, Zhang H, Wang J, Rudolph JE, Yaeger R, Soumerai T, Nissan MH *et al.* (2017) OncoKB: a precision oncology knowledge base. JCO Precis Oncol 1:1-16.

Cibulskis K, Lawrence MS, Carter SL, Sivachenko A, Jaffe D, Sougnez C, Gabriel S, Meyerson M, Lander ES, Getz G (2013) Sensitive detection of somatic point mutations in impure and heterogeneous cancer samples. Nat Biotechnol 31:213-219.

Howitt BE, Shukla SA, Sholl LM, Ritterhouse LL, Watkins JC, Rodig S, Stover E, Strickland KC, D'Andrea AD, Wu CJ *et al*. (2015) Association of polymerase e-mutated and microsatellite-instable 409 endometrial cancers with neoantigen load, number of tumor-infiltrating lymphocytes, and 410 expression of PD-1 and PD-L1. JAMA Oncol 1:1319-1323.

Li, H and Durbin, R (2010) Fast and accurate long-read alignment with Burrows–Wheeler transform. Bioinformatics 26:589-595.

Li, H, Handsaker B, Wysoker A, Fennell T, Ruan J, Homer N, Marth G, Abecasis G, Durbin R, 1000 Genome Project Data Processing Subgroup (2009) The sequence alignment/map format and SAMtools. Bioinformatics 25:2078-2079.

McKenna A, Hanna M, Banks E, Sivachenko A, Cibulskis K, Kernytsky A, Garimella K, Altshuler D, Gabriel S, Daly M *et al*. (2010) The Genome Analysis Toolkit: a MapReduce framework for analyzing next-generation DNA sequencing data. Genome Res. 20:1297-1303.

Petryk N, Kahli M, d'Aubenton-Carafa Y, Jaszczyszyn Y, Shen Y, Silvain M, Thermes C, Chen CL, Hyrien O (2016) Replication landscape of the human genome. Nat Commun 7:10208.

Prentice LM, Miller RR, Knaggs J, Mazloomian A, Hernandez RA, Franchini P et al. (2018). Formalin fixation increases deamination mutation signature but should not lead to false positive mutations in clinical practice. PLoS One 13:e0196434.

Pugh TJ, Amr SS, Bowser MJ, Gowrisankar S, Hynes E, Mahanta LM, Rehm HL, Funke B, Lebo MS (2016) VisCap: inference and visualization of germ-line copy-number variants from targeted clinical sequencing data. Genet Med. 18:712-719.

Ramos AH, Lichtenstein L, Gupta M, Lawrence M.S, Pugh TJ, Saksena G, Meyerson M, Getz G (2015) Oncotator: cancer variant annotation tool. Hum Mutat 36:E2423-2429.

Shen R and Seshan VE (2016) FACETS: allele-specific copy number and clonal heterogeneity analysis tool for high-throughput DNA sequencing. Nucleic Acids Res 44:e131-e131.

Van der Auwera GA, Carneiro MO, Hartl C, Poplin R, Del Angel G, Levy-Moonshine A, Jordan T, Shakir K, Roazen D, Thibault J *et al.* (2013) From FastQ data to high-confidence variant calls: the genome analysis toolkit best practices pipeline. Curr Protoc Bioinformatics 43:11-10.

Wang K, Mingyao Li M and Hakon Hakonarson H (2010) ANNOVAR: functional annotation of genetic variants from high-throughput sequencing data. Nucleic Acids Res 38:e164-e164.

Zhang J, Baran J, Cros A, Guberman JM, Haider S, Hsu J, Liang Y, Rivkin E, Wang J, Whitty B *et al*. (2011) International Cancer Genome Consortium Data Portal--a one-stop shop for cancer genomics data. Database (Oxford) 2011:bar026.
