# Supplementary material for: First description of ultramutated endometrial cancer caused by germline loss-of-function and somatic exonuclease domain mutations in POLE gene
Source: Genet Mol Biol. 2020 Sep 25;43(4):e20200100. doi: 10.1590/1678-4685-GMB-2020-0100 (PMC7521106; doi:10.1590/1678-4685-GMB-2020-0100)
Supplement: Supplementary file 2 [file 1415-4757-GMB-43-4-e20200100-suppl2.pdf]

Genetics and Molecular Biology
https://doi.org/10.1590/1678-4685-GMB-2020-0100

Supplementary Material to “First description of ultramutated endometrial cancer caused by germline loss-of-function and somatic exonuclease domain mutations in *POLE* gene”

Table S1 – Detailed list of the 63 genes used for targeted sequencing.

HUGO Symbol	RefSeq ID	Coding exon count
AKT1	NM_001014431	13
APC	NM_000038	15
ATM	NM_000051	62
ATR	NM_001184	47
AXIN2	NM_004655	10
BARD1	NM_000465	11
BMPR1A	NM_004329	11
BRCA1	NM_007300	23
BRCA2	NM_000059	26
BRIP1	NM_032043	19
CDH1	NM_004360	16
CDK4	NM_000075	7
CDKN2A	NM_000077	3
CHEK2	NM_001005735	15
EGFR	NM_005228	28
EPCAM	NM_002354	9
EXO1	NM_130398	13
FAN1	NM_014967	13
FANCC	NM_000136	14
GALNT12	NM_024642	10
GREM1	NM_013372	1
IGF1R	NM_000875	21
KLLN	NM_001126049	1
MLH1	NM_000249	19
MLH3	NM_001040108	12
MRE11A	NM_005591	19
MSH2	NM_000251	16
MSH3	NM_002439	24
MSH6	NM_000179	10
MUTYH	NM_001128425	16

HUGO Symbol	RefSeq ID	Coding exon count
NBN	NM_002485	16
NTHL1	NM_002528	6
PALB2	NM_024675	13
PCNA	NM_182649	6
PDGFRA	NM_006206	22
PIK3CA	NM_006218	20
PMS1	NM_000534	12
PMS2	NM_000535	15
POLD1	NM_001256849	26
POLE	NM_006231	49
PTEN	NM_000314	9
RAD50	NM_005732	25
RAD51C	NM_058216	9
RAD51D	NM_002878	10
RFC1	NM_001204747	25
RFC2	NM_181471	11
RFC3	NM_002915	9
RFC4	NM_002916	10
RFC5	NM_007370	11
RINT1	NM_021930	15
RPA1	NM_002945	17
RPA2	NM_002946	9
RPA3	NM_002947	4
RPS20	NM_001146227	5
SDHB	NM_003000	8
SDHC	NM_003001	6
SDHD	NM_003002	4
SEC23B	NM_032986	19
SMAD4	NM_005359	11
STK11	NM_000455	9
TGFA	NM_003236	6
TGFBR2	NM_001024847	8
TP53	NM_001276760	8
